# 

**Published:** 1874-05-01

**Authors:** 


No. IX. CHICAGO, MAY 1, 1874. Vol. XV.

### THE Medical Examiner.

### A Semi-Monthly Journal of Medical Sciences.

*TERMS.— Yearly Subscriptions, Three Dollars, in advance. Single Number Fifteen Cents. All Communications should be addressed to Publishers* MEDICAL EXAMINER, 65 *Randolph st., cor. State, Chicago.*

